# Predicting intraoperative hypotension using deep learning with waveforms of arterial blood pressure, electroencephalogram, and electrocardiogram: Retrospective study

**DOI:** 10.1371/journal.pone.0272055

**Published:** 2022-08-09

**Authors:** Yong-Yeon Jo, Jong-Hwan Jang, Joon-myoung Kwon, Hyung-Chul Lee, Chul-Woo Jung, Seonjeong Byun, Han‐Gil Jeong

**Affiliations:** 1 AI Research Team, Medical AI, Co. Ltd., Seoul, Republic of Korea; 2 Department of Biomedical Systems Informatics, Yonsei University College of Medicine, Yongin, Gyeonggi-do, Republic of Korea; 3 Department of Emergency Medicine, Mediplex Sejong Hospital, Incheon, Republic of Korea; 4 Department of Anesthesiology and Pain Medicine, Seoul National University College of Medicine, Seoul National University Hospital, Seoul, Republic of Korea; 5 Department of Psychiatry, Uijeongbu St. Mary’s Hospital, College of Medicine, The Catholic University of Korea, Uijeongbu, Gyeonggi-do, Republic of Korea; 6 Division of Neurocritical Care, Department of Neurosurgery and Neurology, Seoul National University Bundang Hospital, Seoul National University College of Medicine, Seongnam, Republic of Korea; 7 Center for Artificial Intelligence in Healthcare, Seoul National University Bundang Hospital, Seongnam, Republic of Korea; Kuwait College of Science and Technology, KUWAIT

## Abstract

To develop deep learning models for predicting Interoperative hypotension (IOH) using waveforms from arterial blood pressure (ABP), electrocardiogram (ECG), and electroencephalogram (EEG), and to determine whether combination ABP with EEG or CG improves model performance. Data were retrieved from VitalDB, a public data repository of vital signs taken during surgeries in 10 operating rooms at Seoul National University Hospital from January 6, 2005, to March 1, 2014. Retrospective data from 14,140 adult patients undergoing non-cardiac surgery with general anaesthesia were used. The predictive performances of models trained with different combinations of waveforms were evaluated and compared at time points at 3, 5, 10, 15 minutes before the event. The performance was calculated by area under the receiver operating characteristic (AUROC), area under the precision-recall curve (AUPRC), sensitivity and specificity. The model performance was better in the model using both ABP and EEG waveforms than in all other models at all time points (3, 5, 10, and 15 minutes before an event) Using high-fidelity ABP and EEG waveforms, the model predicted IOH with a AUROC and AUPRC of 0.935 [0.932 to 0.938] and 0.882 [0.876 to 0.887] at 5 minutes before an IOH event. The output of both ABP and EEG was more calibrated than that using other combinations or ABP alone. The results demonstrate that a predictive deep neural network can be trained using ABP, ECG, and EEG waveforms, and the combination of ABP and EEG improves model performance and calibration.

## Introduction

Intraoperative hypotension is conventionally defined as a drop in mean arterial pressure (MAP) to less than 65 mmHg during surgery,[1] which is associated with postoperative myocardial infarction, acute kidney injury, and postoperative mortality [2, 3]. Factors affecting blood pressure drop during surgery are multifactorial, including bleeding during surgery, anaesthetics, underlying illnesses, and certain preoperative medications [4, 5]. If intraoperative hypotension could be identified in advance, it could then be addressed quickly and prevented using various measures. This would ultimately improve the postoperative patient outcome. Recently, a randomized clinical trial used a machine-learning-derived early-warning system with certain parameters derived from the arterial pressure wave, resulting in less intraoperative hypotension [6].

The electrocardiogram (ECG) waveform is the most monitored biosignal in operating rooms and intensive care units. Cardiac-rhythm disturbances, myocardial ischemia, and electrolyte disturbances, which can lead to intra-operative hypotension, are reflected in ECG monitoring [7]. Additionally, during surgery, some patients undergo two- or four-channel electroencephalogram (EEG) monitoring procedures, such as the bispectral index measurement, to monitor the depth of sedation or anesthesia [8]. Deep anaesthesia can cause intra-operative hypotension via the substantial inhibition of sympathetic activity, and it can reduce myocardial contractility and systemic vascular resistance. Furthermore, several studies have shown that low bispectral index values during surgery are also associated with postoperative mortality [9–12].

Most previous studies on machine-learning derived early warning algorithms are limited by relying only one a single data source such as arterial pressure waveforms or photoplethysmographs [13–15]. However, hemodynamic changes are also associated with alteration in physiological profiles, including ECG and EEG [16]. In addition, early warning models based on arterial blood pressure (ABP) waveforms necessarily require extensive feature engineering based on proprietary algorithms. In the present study, we hypothesize that combining ABP waveforms with EEG or ECG may better predict intraoperative hypotension. In this study, we trained a deep neural network using waveforms of ABP, ECG, and EEG during surgery without specific hemodynamic parameters derived from a proprietary algorithm.

## Materials and methods

### Objective

The objective of this study was to develop a model to predict intraoperative hypotension using ABP, ECG, and EEG waveforms. Intraoperative hypotension is defined as a drop in mean arterial pressure (MAP) of less than 65 mmHg.

### Dataset

**Fig 1:**
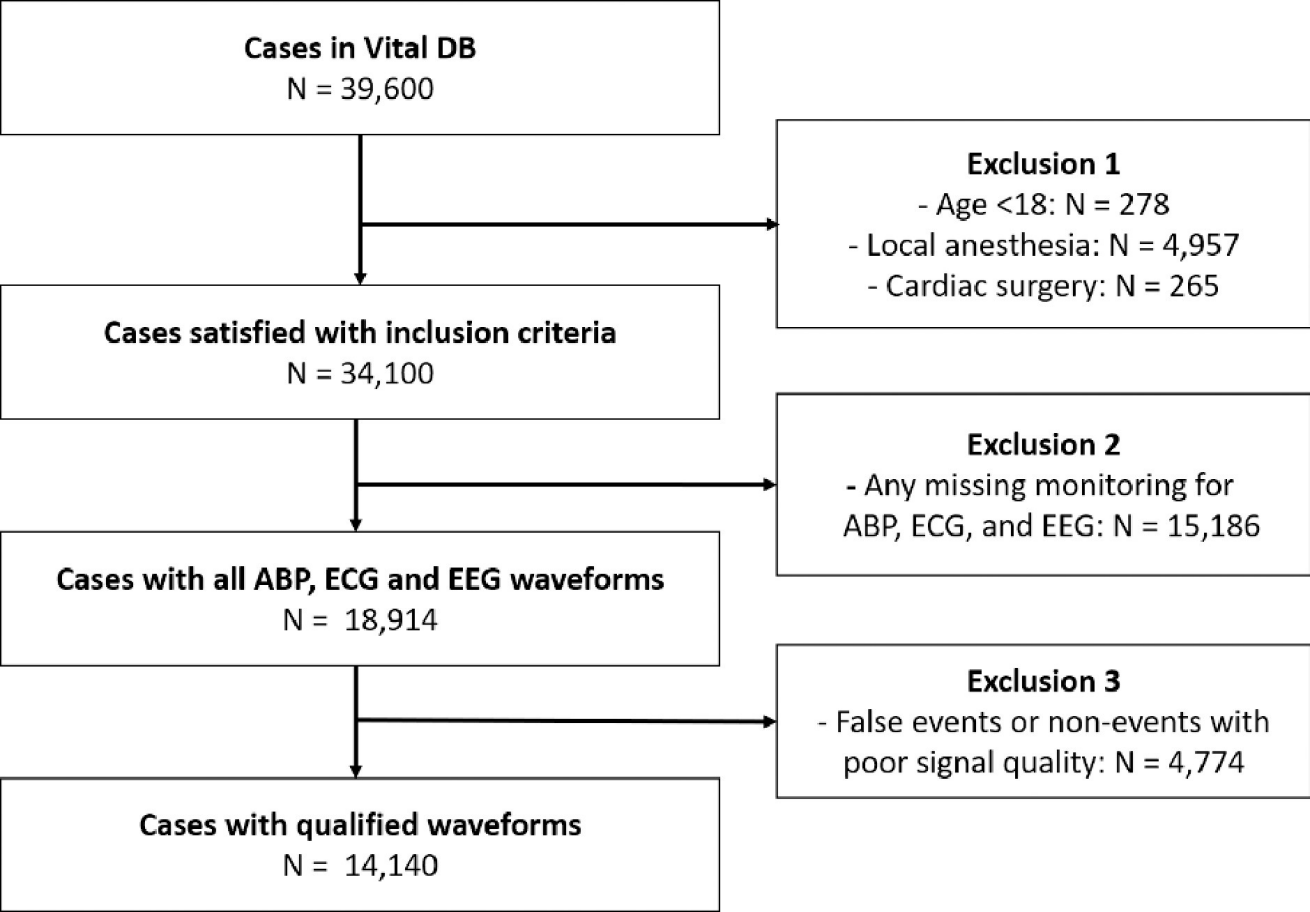
Patient flowchart.

Data were retrieved from VitalDB, a public data repository of vital signs taken during surgeries from January 6, 2005, to March 1, 2014. A data-recording software (Vital Recorder 1.7.4; https://vitaldb.net/vital-recorder) was used to collect the data from 10 operating rooms at Seoul National University Hospital [17]. The inclusion criteria were as follows: (1) adults (age≥18); (2) administered general anaesthesia; and (3) undergone non-cardiac surgery. The exclusion criteria were as follows: (1) any missing monitoring for ABP, ECG, and EEG waveforms; and (2) cases containing false events or non-events due to poor signal quality shown as Fig 1.

### Data selection/Pre-processing

We defined a hypotensive event as a 1-min interval in which a patient sustains a MAP of less than 65 mmHg during surgery. We defined the section in which hypotensive events appear in succession as a hypotensive segment. However, the MAP used to define the event may contain an outlier in some cases because the ABP waveform is noisy and it caused to generates false event or non-event. To remove unreliable case, we used an algorithm named j signal quality index (jSQI) [18], which calculates the ABP waveform’s noise score. Events and non-event cases extracted from noisy ABP waveforms with jSQI 0.8 or less, were excluded. We sampled patient events at ~20-min intervals to minimize potential residual effects from the previous event (see S1 Fig). Then, ABP, ECG, and EEG waveforms of 1-min intervals were collected at 3, 5, 10, and 15 min before each event. For sampling non-events, 30-min segments, in which MAPs per minute was maintained above 75 mmHg, were first extracted, and three samples of each waveform of 1-min intervals were obtained in the middle of the segment. The sampling rates for ABP, ECG, and EEG waveforms were 500, 500, and 128 Hz, respectively. After applying filters with various range of filtering, we decided on the following pre-processing methods. ECG waveforms were pre-processed with a 1–40-Hz band-pass filter and normalized using Z-score. EEG waveforms were pre-processed using a 0.5–50-Hz band-pass filter. ABP waveforms were used without pre-processing.

We split the data based on the number of cases into training, validation, and test datasets in a 6:1:3 ratio, while preventing the distribution of samples derived from a single case into different datasets. The number of cases in Table 2 at every time point is not equivalent. This is because, when a hypotensive event occurs at the beginning of the signal data (within 15 minutes), sampling of ABP, ECG, and EEG waveforms is possible only at some time points among the data segments 3, 5, 10, and 15 minutes before the event, depending on the timing of the occurrence.

### Model development

In this study, we developed a model based on ResNet [19], which is a popular one in image classification models. ResNet solves the gradient vanishing problem as layers deepen through residual learning using skip connections that add the input to the output after several layers. We tuned the ResNet for our purpose to extract important features from each waveform. Our model consists of three ResNets and one classifier.

**Fig 2:**
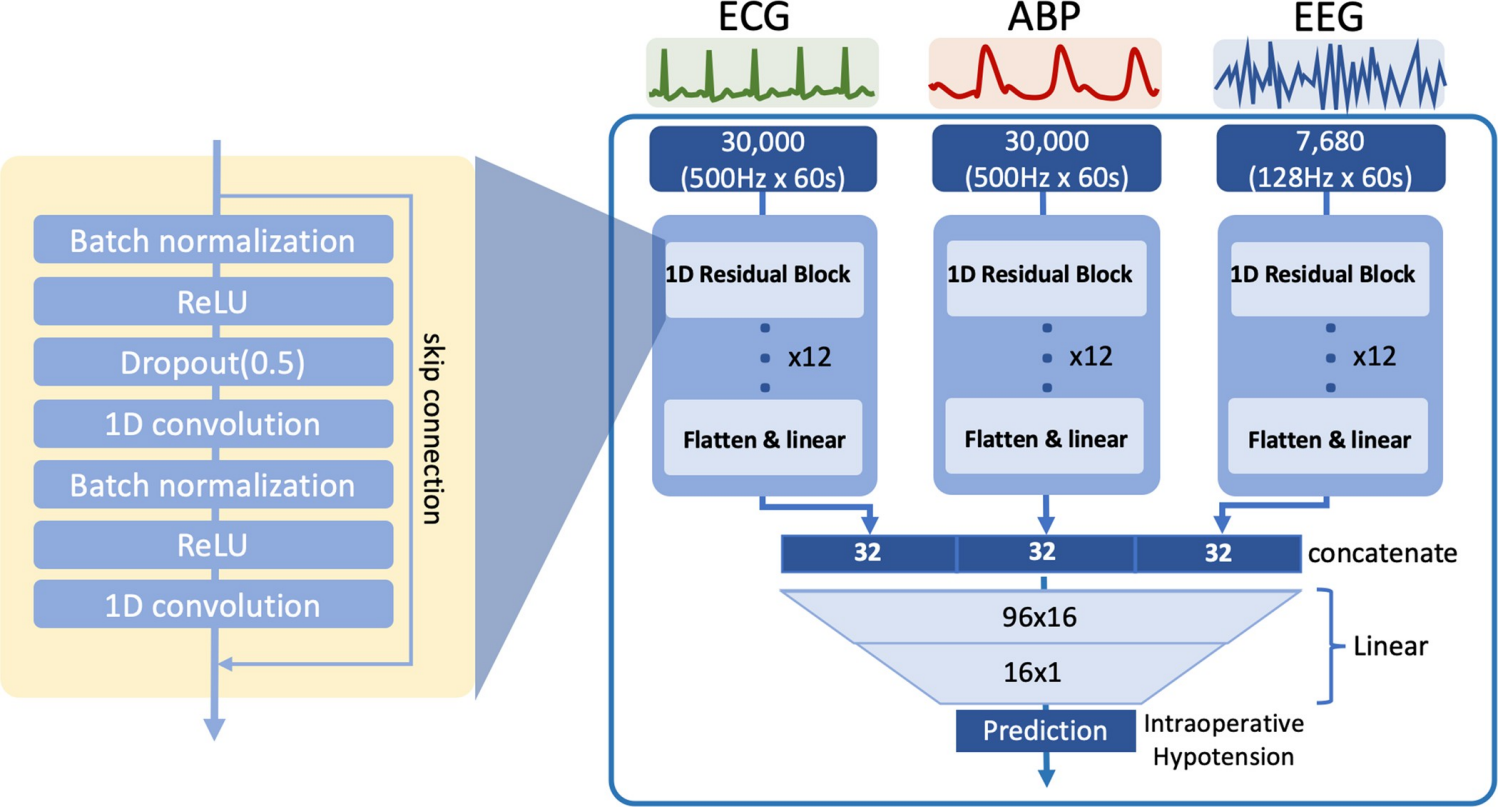
Architecture of the hypotension risk prediction model using multiple waveforms.

Fig 2 shows an example of our model architecture. Each ResNet has a single encoder block, multiple residual blocks, and a fully connected layer. The encoder block consisted of a convolutional neural network (CNN) with a max pooling function. CNN is a deep-learning method for analyzing image data. It can learn to extract positional and morphological information from data using filters, which are parameters that extract features by moving at assigned intervals called strides. One-dimensional data such as audio and biosignal data can also be analyzed with one-dimensional CNN used in this study that was designed for one-dimensional sequential data. Each residual block has two sets of CNNs with batch normalization, dropout, and rectified linear-unit activation functions. The input and output of the residual block are summed by skip-connection, which is known to improve the efficiency of a training model consisting of many layers. Because we use a combination of waveforms to predict intraoperative hypotension, the models have either a single network or multiple networks depending on the number of biosignals. The output of each network is concatenated and is passed to a classifier with two fully connected layers and a sigmoid function for predicting intraoperative hypotension using concatenated outputs derived from every network.

Each ResNet contains 12 residual blocks and one linear layer. Detailed hyperparameter settings were described in S1 Table. We set that the output size of ResNet for each biosignal is 32. In our models, Binary cross entropy is used as the loss function, and Adam is used as the optimizer. Through some preliminary experiments, the filter size of each waveform ABP, ECG, and EEG was set to be 15, 15, and 7, respectively. Stride was set as one for all CNN layers, the dropout was 0.5, and the learning rate was 0.0001. We set the epoch to 100, and stop the process of training the model early when there was no loss reduction on the validation set over five epochs. We choose the final model with the lowest loss. The model at 68th epoch in the training step is selected. We have a limitation for the hyper parameter tuning due to extensive experiments and computing power.

### Experimental setup

The performance of the model was evaluated using the test dataset. AUROC, AUPRC, sensitivity, and specificity were calculated. The optimal cut-off value was calculated to minimize the difference between the sensitivity and specificity. Confidence intervals (CI) for each value were calculated using the exact binomial confidence limits. Our task was performed on a workstation equipped with an Intel Xeon Silver 4114 processor, 128-GB RAM, and two NVIDIA RTX TITAN graphics processing units.

### Correlation analysis between hypotension prediction and actual occurrence

All model-output values for the event and non-event samples in the test dataset were gathered and segmented into model-output bins. In each bin, the percentage of event samples was the rate of events occurring in given time.

#### Post hoc analysis for model comparison

We developed and tested our model by sampling hypotensive events with at least 20-min intervals to exclude the residual effect of the previous hypotensive event. To compare the performance of our model with that previously reported by Hatib et al. [14], we adopted the same sampling strategy, which captures every hypotensive event in the test dataset. Then, we calculated and presented AUROC and AUPRC in our models using only ABP and a combination of ABP and EEG.

## Results

### Dataset characteristics

Among the eligible cases, the mean age was 58.7 years. Males comprised 50.6% (n = 6,850) of the cases. Total anaesthesia duration was 240±113 min, and total surgery duration was 180±105 min. Emergent operation consisted of 7.2% (n = 969) of surgeries. 16% (n = 2,061) of cases were American Society of Anesthesiologists (ASA) classifications of three or more, which means severe pre-anaesthesia medical comorbidities occurred. The patient’s characteristics were comparable between the training and testing datasets in Table 1. There is no significant difference among train, test and validation datasets except weight (p-value < 0.05).

**Table 1:** Dataset characteristics. Overall missing values are below 1%. ASA, American Society of Anesthesiologists classification.

Variables	Total (N = 14,140)	Train (N = 9,898)	Test (N = 4,242)
Age	58.8 ± 14.9	58.8 ± 14.9	58.8 ± 14.8
Male sex	7,137 (50.5%)	4,979 (50.3%)	2,158 (50.9%)
Height, cm	162.0 ± 9.7	162.0 ± 9.7	162.2 ± 9.7
Weight, kg	61.7 ± 11.9	61.6 ± 11.9	62.0 ± 11.9
Anesthesia duration, min	237.3 ± 113.5	237.4 ± 113.2	237.2 ± 114.2
Surgery duration, min	177.2 ± 105.0	177.2 ± 104.4	177.2 ± 106.6
Emergent operation	1027 (7.3%)	725 (7.3%)	302 (7.1%)
ASA			
1	3184 (23.4%)	2196 (23.1%)	988 (24.3%)
2	8250 (60.7%)	5821 (61.2%)	2429 (59.7%)
3–6	2157 (15.9%)	1502 (15.8%)	655 (16.1%)
Hypertension	2144 (15.2%)	1504 (15.2%)	640 (15.1%)
Diabetes mellitus	1114 (7.9%)	792 (8.0%)	322 (7.6%)
Cardiac disease	677 (4.8%)	470 (4.7%)	207 (4.9%)
Renal disease	568 (4.0%)	397 (4.0%)	171 (4.0%)
Liver disease	703 (5.0%)	508 (5.1%)	195 (4.6%)
Hemoglobin, g/dL	12.8 ± 1.9	12.8 ± 1.9	12.8 ± 1.9
Platelet, x10^3^/uL	241.0 ± 83.8	241.0 ± 83.1	240.9 ± 85.5
Blood urea nitrogen, mg/dL	16.9 ± 10.6	16.9 ± 10.6	16.8 ± 10.5
Creatinine, mg/dL	1.1 ± 1.6	1.1 ± 1.6	1.1 ± 1.5
Albumin, g/dL	4.0 ± 0.5	4.0 ± 0.5	4.0 ± 0.5

The number of cases and ABP, ECG, and EEG waveforms that were split into training, validation, and test sets are described in Table 2. In brief, waveforms were sampled 3, 5, 10, and 15 min before the occurrence of hypotension for events and were sampled in the middle of 30-min segments of normal arterial pressure for non-events. The data below are expressed as mean±SD (median and [25th–75th] quartiles) minutes. The dataset contained 84,435 hypotensive segments (total duration, 287,417 min) and 169,189 non-hypotensive segments (total duration, 2,048,109 min). There were 20.3±33.7 (10 [3–23]) hypotensive events per patient, representing 13.4±17.1% (6.8 [2.1–17.3]) of each patient’s monitoring time. Each hypotensive segment lasted 6.0±6.4 (4 [2–8]) min.

**Table 2:** Data composition.

	Train		Validation		Test	
**Time to event**	Event samples (cases) [recurrent events]	Non-event samples (cases)	Event samples (cases) [recurrent events]	Non-event samples (cases)	Event samples (cases) [recurrent events]	Non-event samples (cases)
**3 min**	16,149 (5,814) [10,335]	56,277 (6,269)	2,835 (1,004) [1,831]	9,548 (1,040)	8,403 (2,925) [5,478]	27,204 (3,103)
**5 min**	16,297 (5,823) [10,474]	55,914 (6,242)	2,701 (949) [1,752]	9,757 (1,065)	8,346 (2,953) [5,393]	27,358 (3,105)
**10 min**	15,627 (5,641) [9,986]	56,351 (6,283)	2,709 (950) [1,759]	9,385 (1,045)	8,026 (2,877) [5,149]	27,293 (3,084)
**15 min**	15,494 (5,480) [10,14]	55,246 (6,226)	2,383 (901) [1,482]	9,684 (1,049)	7,153 (2,703) [4,450]	28,099 (3,137)

### Hypotension prediction performance depending on a set of combinations of waveforms

The prediction performance was evaluated using area under the receiver operating characteristic curve (AUROC), area under the precision-recall curve (AUPRC), sensitivity, and specificity at various time points and waveform compositions. Among models using a single waveform, ABP was much better than ECG or EEG when predicting intraoperative hypotension. Of the models using multiple waveforms, the combination of ABP and EEG waveforms was generally the best, outperforming models that used only ABP in Table 3 (Also, S2 Table). The representative attention map of the model using both ABP and EEG is provided in S2 Fig. Our model generally performs better when predicting the recurrent events than the first occurring events (S3 Table).

**Table 3:** Area under the Receiver-operating Characteristic Curve, Area under the Precision-Recall Curve, Sensitivity, and Specificity of our model in predicting intraoperative hypotension. Value (95% confidence interval); AUROC, area under the receiver operating characteristic; AUPRC, area under the precision-recall curve; ABP, arterial blood pressure; ECG, electrocardiogram; EEG, electroencephalogram.

Waveforms	AUROC	AUPRC	Sensitivity	Specificity	Threshold
**Time to event: 3 min**					
ABP	0.968 (0.966–0.970)	0.939 (0.935–0.943)	0.914 (0.911–0.918)	0.916 (0.909–0.921)	0.54
ECG	0.634 (0.627–0.640)	0.339 (0.329–0.348)	0.607 (0.601–0.613)	0.593 (0.583–0.604)	0.51
EEG	0.557 (0.550–0.563)	0.286 (0.278–0.294)	0.508 (0.502–0.514)	0.576 (0.566–0.587)	0.51
ABP + ECG	0.967 (0.965–0.970)	0.936 (0.932–0.940)	0.912 (0.909–0.916)	0.913 (0.907–0.919)	0.62
ABP + EEG	**0.970 (0.968–0.972)**	**0.943 (0.939–0.946)**	**0.917 (0.914–0.920)**	**0.917 (0.911–0.923)**	0.42
ECG + EEG	0.636 (0.629–0.643)	0.340 (0.331–0.351)	0.603 (0.597–0.609)	0.598 (0.588–0.609)	0.47
ABP + ECG + EEG	0.957 (0.954–0.960)	0.926 (0.921–0.931)	0.903 (0.900–0.907)	0.905 (0.898–0.911)	0.30
**Time to event: 5 min**					
ABP	0.930 (0.927–0.934)	0.873 (0.867–0.878)	0.859 (0.855–0.863)	0.856 (0.848–0.863)	0.43
ECG	0.652 (0.645–0.658)	0.359 (0.350–0.369)	0.608 (0.602–0.614)	0.614 (0.604–0.625)	0.55
EEG	0.581 (0.574–0.588)	0.301 (0.293–0.310)	0.579 (0.573–0.585)	0.528 (0.517–0.538)	0.53
ABP + ECG	0.929 (0.925–0.933)	0.874 (0.868–0.879)	0.854 (0.850–0.858)	0.852 (0.844–0.860)	0.48
ABP + EEG	**0.935 (0.932–0.938)**	**0.882 (0.876–0.887)**	**0.860 (0.856–0.864)**	**0.858 (0.851–0.866)**	0.42
ECG + EEG	0.646 (0.639–0.653)	0.363 (0.354–0.373)	0.614 (0.608–0.620)	0.602 (0.591–0.612)	0.57
ABP + ECG + EEG	0.926 (0.923–0.930)	0.867 (0.861–0.873)	0.849 (0.845–0.854)	0.852 (0.844–0.859)	0.35
**Time to event: 10 min**					
ABP	0.892 (0.887–0.897)	0.814 (0.807–0.822)	0.807 (0.802–0.811)	0.803 (0.794–0.812)	0.43
ECG	0.659 (0.653–0.666)	0.364 (0.353–0.373)	0.611 (0.605–0.617)	0.612 (0.600–0.623)	0.52
EEG	0.584 (0.577–0.592)	0.297 (0.289–0.306)	0.551 (0.545–0.556)	0.529 (0.518–0.541)	0.52
ABP + ECG	0.867 (0.862–0.872)	0.791 (0.783–0.798)	0.792 (0.787–0.797)	0.792 (0.782–0.801)	0.40
ABP + EEG	**0.898 (0.893–0.902)**	**0.819 (0.812–0.827)**	0.813 (0.808–0.817)	**0.811 (0.802–0.820)**	0.46
ECG + EEG	0.645 (0.638–0.652)	0.347 (0.337–0.358)	0.607 (0.602–0.613)	0.579 (0.568–0.591)	0.49
ABP + ECG + EEG	0.895 (0.891–0.900)	0.817 (0.809–0.824)	**0.814 (0.809–0.818)**	0.788 (0.778–0.797)	0.48
**Time to event: 15 min**					
ABP	0.889 (0.884–0.894)	0.803 (0.795–0.810)	0.801 (0.796–0.806)	0.803 (0.794–0.812)	0.43
ECG	0.640 (0.634–0.648)	0.306 (0.297–0.315)	0.595 (0.589–0.601)	0.612 (0.600–0.623)	0.57
EEG	0.577 (0.570–0.585)	0.260 (0.252–0.269)	0.584 (0.578–0.590)	0.529 (0.518–0.541)	0.51
ABP + ECG	0.874 (0.868–0.879)	0.784 (0.776–0.792)	0.793 (0.788–0.798)	0.792 0.782–0.801)	0.38
ABP + EEG	**0.894 (0.889–0.899)**	**0.808 (0.801–0.816)**	**0.806 (0.801–0.810)**	**0.811 (0.802–0.820)**	0.33
ECG + EEG	0.623 (0.616–0.630)	0.292 (0.283–0.301)	0.595 (0.589–0.601)	0.579 (0.568–0.591)	0.53
ABP + ECG + EEG	0.868 (0.862–0.874)	0.778 (0.769–0.786)	0.793 (0.788–0.797)	0.788 (0.778–0.797)	0.47

**Fig 3:**
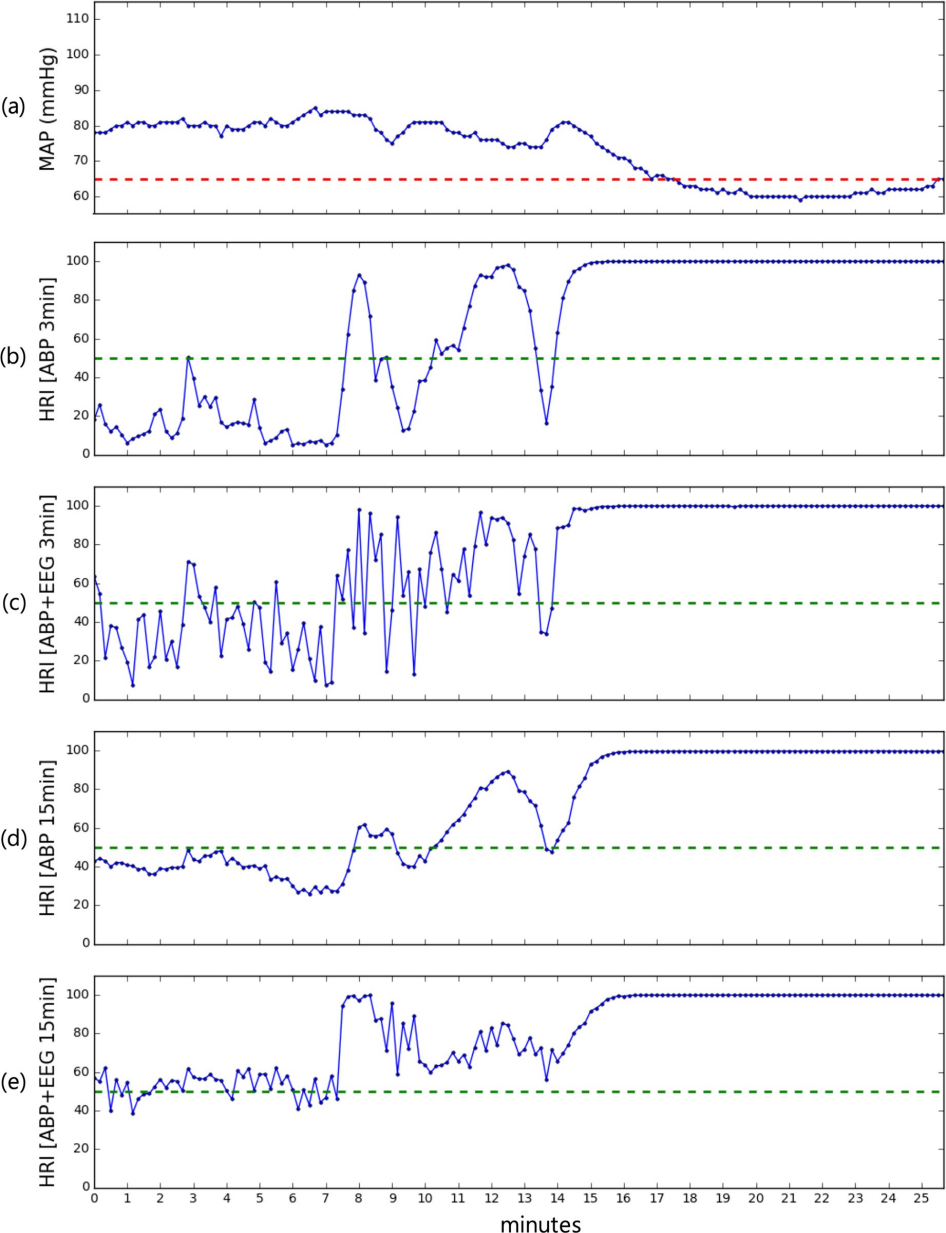
Illustrative patient record demonstrating the trajectory of algorithm outputs from different combinations of inputs (time and signals) and mean arterial pressure. (a) Trajectory of MAP. Red dash, 65 mmHg. (b) HRI from ABP waveform and (c) a combination of ABP and EEG for predicting event 3-min before, respectively. (d) HRI from ABP waveform, and e a combination of ABP and EEG waveforms for predicting event 15 min before. MAP, mean arterial pressure; HRI, hypotension risk index; ABP, arterial blood pressure; EEG, electroencephalogram.

Fig 3 illustrates the trajectory of MAP and the hypotension risk index (HRI is the model output × 100) using a typical example of intraoperative hypotension. In this example, although MAP was stable in the 70- to 85-mmHg range, the HRI required to predict the event 3-min later using ABP (HRI ABP 3 min) increased sharply from approximately 20 to 95, which was 3 min before the hypotensive event. This remained until the event occurred. The HRI using both ABP and EEG (HRI ABP+EEG 3 min) showed similar but more fluctuating predictions. The model outputs predicting hypotension 15 min before showed more robust prediction with both ABP and EEG (HRI ABP+EEG 15 min) than with ABP only (HRI ABP 15 min), which had higher values and stiff increments over 95% around 10-min before the hypotensive event.

### Model output and frequency of hypotension analysis

**Fig 4:**
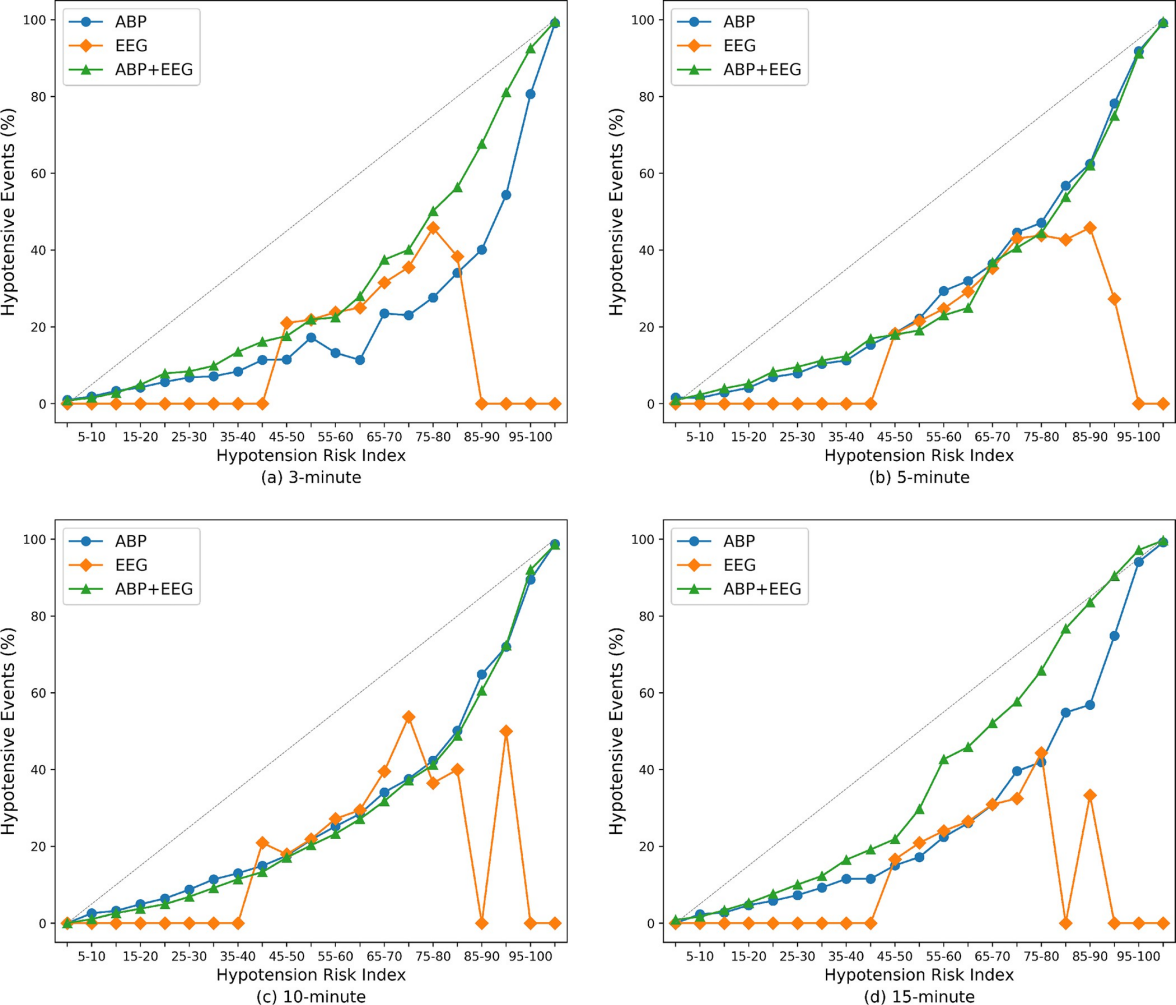
Actual occurrence of hypotensive events according to the Hypotension Risk Index. (a–d) Actual occurrence of hypotensive events according to the hypotension risk indices to predict the events 3, 5, 10, and 15 min before, respectively. ABP, arterial blood pressure; EEG, electroencephalogram.

Fig 4 compares the actual occurrences of hypotension in each bin of the HRI among models using ABP, EEG, or both. Interestingly, the model using both ABP and EEG demonstrates a more linear relationship when predicting hypotensive events, particularly 3 and 15 min before the events. The model using ABP and EEG is more calibrated than are the others having different waveform compositions. The calibration effect in the model using both ABP and EEG is more prominent in the middle-to-high values (HRI ranged 40–95) of the model outputs. The calibration effects using other combinations of each waveform are shown in S3 Fig. The performance of our algorithm on consecutively sampled waveforms with 1-minute interval over the entire duration of a surgical procedure was shown in S4 Table and Fig 4.

#### Post hoc analysis for model comparison

We further tested our model using same-event sampling methods, previously described by Hatib et al. [14], upon which the proprietary Hypotension Prediction Index algorithm is based. In our main analysis, we sampled hypotensive events with at least a 20-min interval to exclude the residual effect of the previous event. In the post hoc analysis, we rebuilt the test set by capturing every hypotensive event at 1-min intervals during surgery to make them comparable with the previous study of Hatib et al. (see S5 Table). Our model demonstrated performance measures similar to the Hypotension Risk Index. The addition of EEG to ABP also improved the model performance at all time points (see Table 4 and S6 Table). The model calibration analysis showed nearly linear associations between model outputs and rates of occurrence of intraoperative hypotension (see S3 Fig).

**Table 4:** Model performance metrics in post hoc analysis. AUROC, Area under the Receiver Operating Characteristic Curve; AUPRC, Area under the Precision-Recall Curve.

	AUROC		AUPRC	
Time to event	ABP	ABP + EEG	ABP	ABP + EEG
**3 min**	0.988 (0.987–0.988)	0.990 (0.989–0.990)	0.994 (0.994–0.994)	0.995 (0.995–0.996)
**5 min**	0.975 (0.974–0.976)	0.976 (0.975–0.976)	0.988 (0.988–0.989)	0.989 (0.989–0.989)
**10 min**	0.945 (0.943–0.946)	0.948 (0.946–0.949)	0.975 (0.974–0.976)	0.976 (0.976–0.977)
**15 min**	0.934 (0.932–0.936)	0.937 (0.936–0.939)	0.967 (0.966–0.968)	0.969 (0.968–0.970)

## Discussions

We developed a deep-learning model to predict intraoperative hypotension from different sets of combinations of ABP, ECG, and EEG waveforms using high-fidelity monitoring data taken over 3-million min from 14,140 patients. The model performance was better in the model using both ABP and EEG waveforms than in all other models at all time points (3, 5, 10, and 15 min). The combination of ABP and EEG waveforms was also beneficial when calibrating model outputs to better reflect actual occurrences of hypotensive events.

Several studies have developed models based on machine learning for the prediction of hemodynamic instability. Lin et al. developed an artificial neural-network model that predicted postinduction hypotension with 82.3% accuracy [20]. Noninvasive features used in the model were commonly used standard variables and were readily retrievable in all anaesthesia records, including 11 patient-related, 2 surgical, and 5 aesthetic variables. Convertino et al. applied novel feature-extraction and machine-learning techniques to plethysmography waveforms to identify patients who were developing cardiac instability [21]. Unfortunately, these approaches do not allow for real-time prediction of hemodynamic instability. In a recent work, Chen et al. predicted hypotension in hemodialysis patients using deep learning [22]. They utilized demographic, clinical, and laboratory data to develop a prediction model. The AUROC in this study was only 0.65, which suggests that it is required to use waveform data for predicting hypotension event using deep learning. Moreover, Davies et al. verified the Edwards Hypotension Prediction Index software in which the AUROCs were 0.92, 0.89, and 0.88 at 5, 10, and 15 minutes before hypotensive event, respectively.

The Hypotension Prediction Index (a commercial hypotension prediction algorithm) calculates various hemodynamic parameters and their combinations and uses them as model features for machine learning. However, for calculating the Hypotension Prediction Index, algorithms incorporated in commercial sensors (e.g., FloTrac and CO-Trek) should be used. In contrast, we utilized a deep-learning architecture that automatically extracts diverse features from waveform data, demonstrating good performance when predicting hypotension. The predictive ability of our model is similar to that seen in the derivation cohorts of the proprietary Hypotension Prediction algorithm [14]. Another machine-learning algorithm was constructed to predict the occurrence of hypotension within 10 min after induction of general anaesthesia [23]. Using a combination of variables, such as comorbidity, preoperative vital signs and medication, the AUROC was 0.76 with a gradient boosting model. Although our algorithm used a more conservative definition of hypotension (<65 mmHg vs. <55 mmHg) and did not depend on other clinical parameters, the predictive performance 10 min before IOH was excellent with AUROC 0.898. This suggests deep learning approaches can benefit from detection of clinically imperceptible and subtle changes from multiple heterogeneous biosignals.

In this study, adding EEG to the ABP waveform improved the model performance at all time points. Intraoperative hypotension is caused either by (a combination of) a reduction in either cardiac preload or afterload or by an impairment in cardiac contractility. Deeper levels of anaesthesia may make patients more susceptible to intraoperative hypotension by reducing sympathetic tone, myocardial contractility, and vascular resistance [23, 24]. On the other hand, EEGs reflect cerebral blood flow and recognize subclinical brain ischemia [25–27]. Thus, hemodynamic instability before blood pressure drop can cause relative hypoperfusion of the brain and may result in subtle changes in EEG activity. This effect can be more prominent when using volatile anaesthetics, which can impair cerebral blood flow autoregulation [28].

Using both EEG and ABP provided more calibrated estimates of occurrence of hypotension than using ABP alone. Although recent advances in deep learning have improved neural-network accuracy, modern neural networks are usually not well-calibrated [29]. A real-time decision-making system, including hypotension prediction, should provide a calibrated confidence measure and its prediction, because final clinical decisions should be made by doctors in charge. The effect of adding EEG to the ABP on calibration was more prominent in the range of model output between 45 and 90, which is above the thresholds of binary classification.

The addition of ECG did not provide additional improvement in the model performance compared with the model using only ABP. In contrast to that from the EEG, most hemodynamic information from the ECG could also be reflected in the ABP waveforms. The electrical waves of the heart obtained through two skin electrodes may not have been more informative than the arterial pressure waves gathered directly within the vessels. However, considering that the subjects in the present study were people who had non-cardiac surgery and most of them had elective surgery, the importance of ECG is likely to be higher in other populations with high rates of emergency operation or high risk of intraoperative myocardial infarction.

Although our algorithm was trained with data from anesthetized patients, this approach could be applied to patients with critical illness such as septic shock and coronavirus disease of 2019 (COVID-19) with further validation and fine-tuning. Hypotension and shock have been observed in a subgroup of patients with severe COVID-19 with possible cytokine storm syndrome [30]. If the risk of hypotension can be predicted with our algorithm, several therapeutic measures, including vasopressors, steroids and novel immunotherapies may be applied earlier and improve outcomes in patients with severe COVID-19.

This study had several limitations. Prospective and external validation are required because validation was performed only with retrospective data and the model may be overfitted in a specific pattern shared at that time. We used data exclusively from anesthetized patients to train our model, which may limit its performance in other clinical settings, such as an intensive care unit. As two-lead ECG and one-channel BIS–EEG waveforms were used in our model, high resolution EEGs or multi-lead ECGs may provide superior performance to our model. For the sake of accuracy, we inevitably built our models based only on data of clear hypotension (MAP < 65 mmHg) and non-hypotension (MAP > 75 mmHg) data, as two easily separable and mutually exclusive label is important for dichotomous classification. Events were extracted at minimum intervals of 20 min, but it is possible that the residual effects of preceding hypotensive events were further learned at waveforms predicting recurrent events. Also, we extracted features from all waveforms by only applying a 1D residual network and used those features to the IOH prediction. As more important features can possibly be extracted by using specific networks for different waveforms, it is further required to learn with different network architectures that is suitable to individual waveforms. These approaches may improve the performance of deep learning models to predict IOH.

## Conclusion

Our deep-learning model trained with waveforms of ABP, EEG, and ECG demonstrated good performance in predicting intraoperative hypotension in patients undergoing non-cardiac surgery. Without specific algorithms for feature extraction, our deep-learning model using raw ABP waveforms showed a high predictive performance. The combination of EEG and ABP may confer enhancements of model performance and calibration of hypotension risk indices.

## Supporting information

S1 Fig Illustrative description of hypotensive events, segments, and sampled hypotensive events. (DOCX)

S2 Fig Representative attention map using gradient-weighted Class Activation Mapping according to Hypotension Risk Index. The hypotension risk index derived from the model to predict hypotension 10-min before using a combination of ABP and EEG waveforms was 66. (a) Attention map of ABP in the whole interval. (b) Magnification of the interval with maximum attention. (c) Attention map for EEG in the whole interval. ABP, arterial blood pressure; EEG, electroencephalogram. We tried to understand our model’s internal operation and to interpret the results of the model using the Gradient-weighted Class Activation Mapping (GradCAM) method.
(DOCX)

S3 Fig Actual occurrence of hypotensive events according to the Hypotension Risk Index for all combinations of waveforms. (a–d) Actual occurrence of hypotensive events according to the hypotension risk indices to predict the events 3, 5, 10, and 15 min before, respectively. ABP, arterial blood pressure; EEG, electroencephalogram; ECG, electrocardiogram.
(DOCX)

S4 Fig The representative cases of prediction over the actual duration of a surgical procedure. PPV, positive predictive value; NPV, negative predictive value; MAP, mean arterial pressure; ABP, arterial blood pressure.
(DOCX)

S1 Table Hyperparameter setting for intraoperative hypotension prediction model. The models have 12 residual blocks. The model using ECG and model using blood pressure was set as same hyperparameter. The model using EEG has a different kernel size.
(DOCX)

S2 Table Statistical significance of comparison of models using different combinations of waveforms. P-values were calculated with DeLong’s method and corrected using Bonferroni’s method.
(DOCX)

S3 Table Difference in model performance between the first event and recurrent events. (DOCX)

S4 Table The model performance on consecutively sampled waveforms with 1-minute interval over the entire surgical procedures in 100 randomly selected cases. (DOCX)

S5 Table Comparison of test dataset between the main and post hoc analysis. (DOCX)

S6 Table Post hoc analysis: Statistical significance of comparison of models using different combinations of waveforms. P-values were calculated with DeLong’s method and corrected using Bonferroni’s method.
(DOCX)
