# Supplementary material for: Predicting intraoperative hypotension using deep learning with waveforms of arterial blood pressure, electroencephalogram, and electrocardiogram: Retrospective study
Source: PLoS One. 2022 Aug 9;17(8):e0272055. doi: 10.1371/journal.pone.0272055 (PMC9362925; doi:10.1371/journal.pone.0272055)
Supplement: S1 Fig — (DOCX) [file pone.0272055.s001.docx]

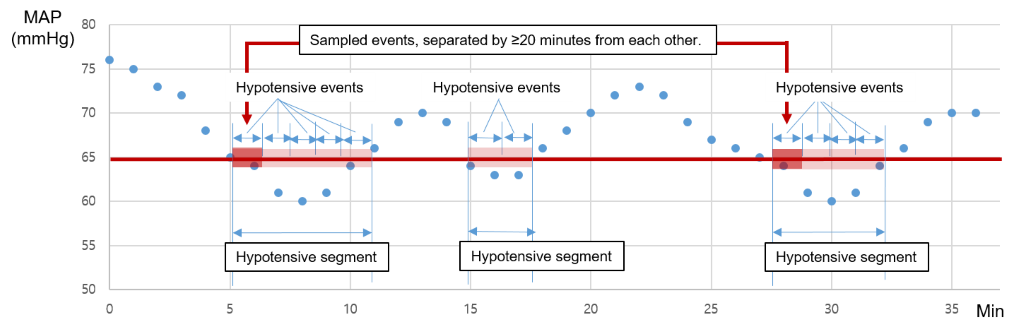


**Supplemental Figure 1.** Illustrative description of hypotensive events, segments, and sampled hypotensive events.
