# Supplementary material for: Predicting intraoperative hypotension using deep learning with waveforms of arterial blood pressure, electroencephalogram, and electrocardiogram: Retrospective study
Source: PLoS One. 2022 Aug 9;17(8):e0272055. doi: 10.1371/journal.pone.0272055 (PMC9362925; doi:10.1371/journal.pone.0272055)
Supplement: S2 Fig — The hypotension risk index derived from the model to predict hypotension 10-min before using a combination of ABP and EEG waveforms was 66. (a) Attention map of ABP in the whole interval. (b) Magnification of the interval with maximum attention. (c) Attention map for EEG in the whole interval. ABP, arterial blood pressure; EEG, electroencephalogram. We tried to understand our model’s internal operation and to interpret the results of the model using the Gradient-weighted Class Activation Mapping (GradCAM) method. (DOCX) [file pone.0272055.s002.docx]

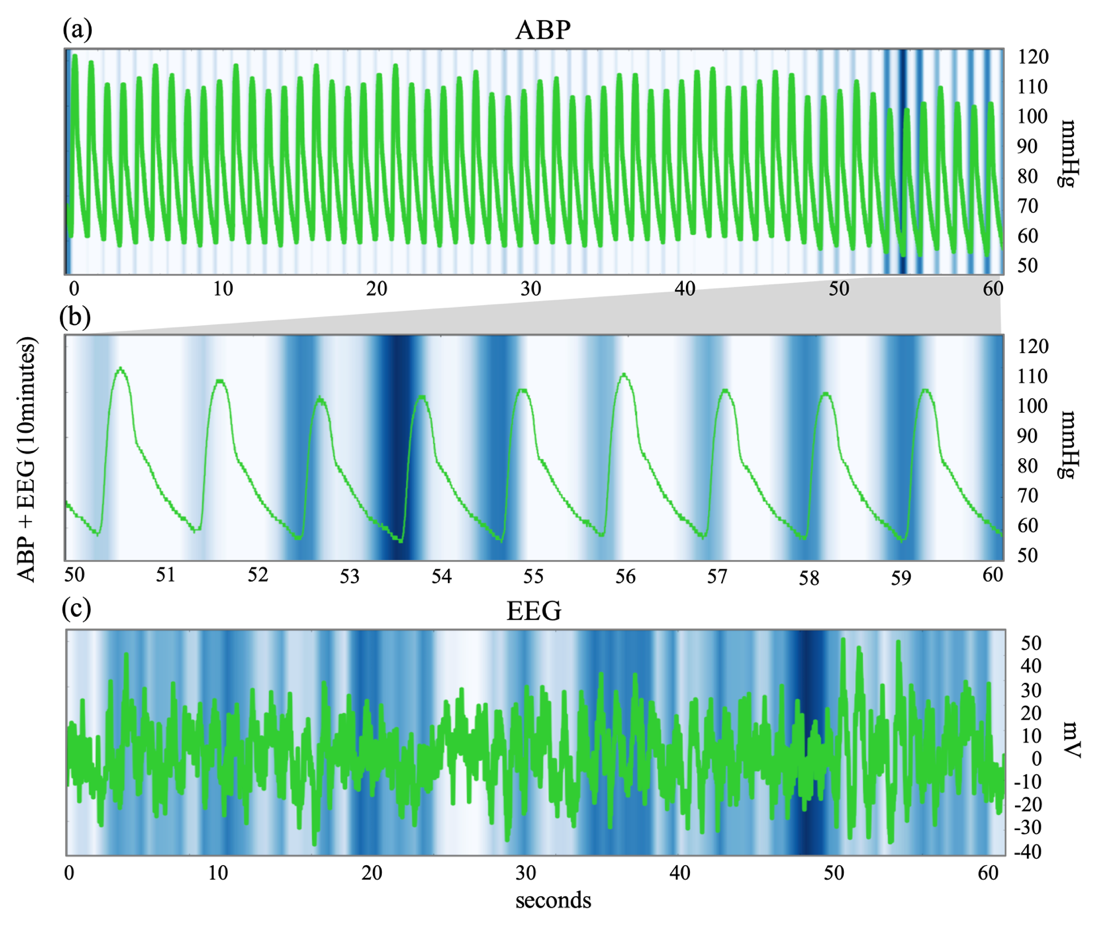


**Supplemental Figure 2.** Representative Attention Map using Gradient-weighted Class Activation Mapping according to Hypotension Risk Index. The hypotension risk index derived from the model to predict hypotension 10-min before using a combination of ABP and EEG waveforms was 66. (a) Attention map of ABP in the whole interval. (b) Magnification of the interval with maximum attention. (c) Attention map for EEG in the whole interval. ABP, arterial blood pressure; EEG, electroencephalogram. We tried to understand our model’s internal operation and to interpret the results of the model using the Gradient-weighted Class Activation Mapping (GradCAM) method.
