# Supplementary material for: Predicting intraoperative hypotension using deep learning with waveforms of arterial blood pressure, electroencephalogram, and electrocardiogram: Retrospective study
Source: PLoS One. 2022 Aug 9;17(8):e0272055. doi: 10.1371/journal.pone.0272055 (PMC9362925; doi:10.1371/journal.pone.0272055)
Supplement: S3 Fig — (a–d) Actual occurrence of hypotensive events according to the hypotension risk indices to predict the events 3, 5, 10, and 15 min before, respectively. ABP, arterial blood pressure; EEG, electroencephalogram; ECG, electrocardiogram. (DOCX) [file pone.0272055.s003.docx]

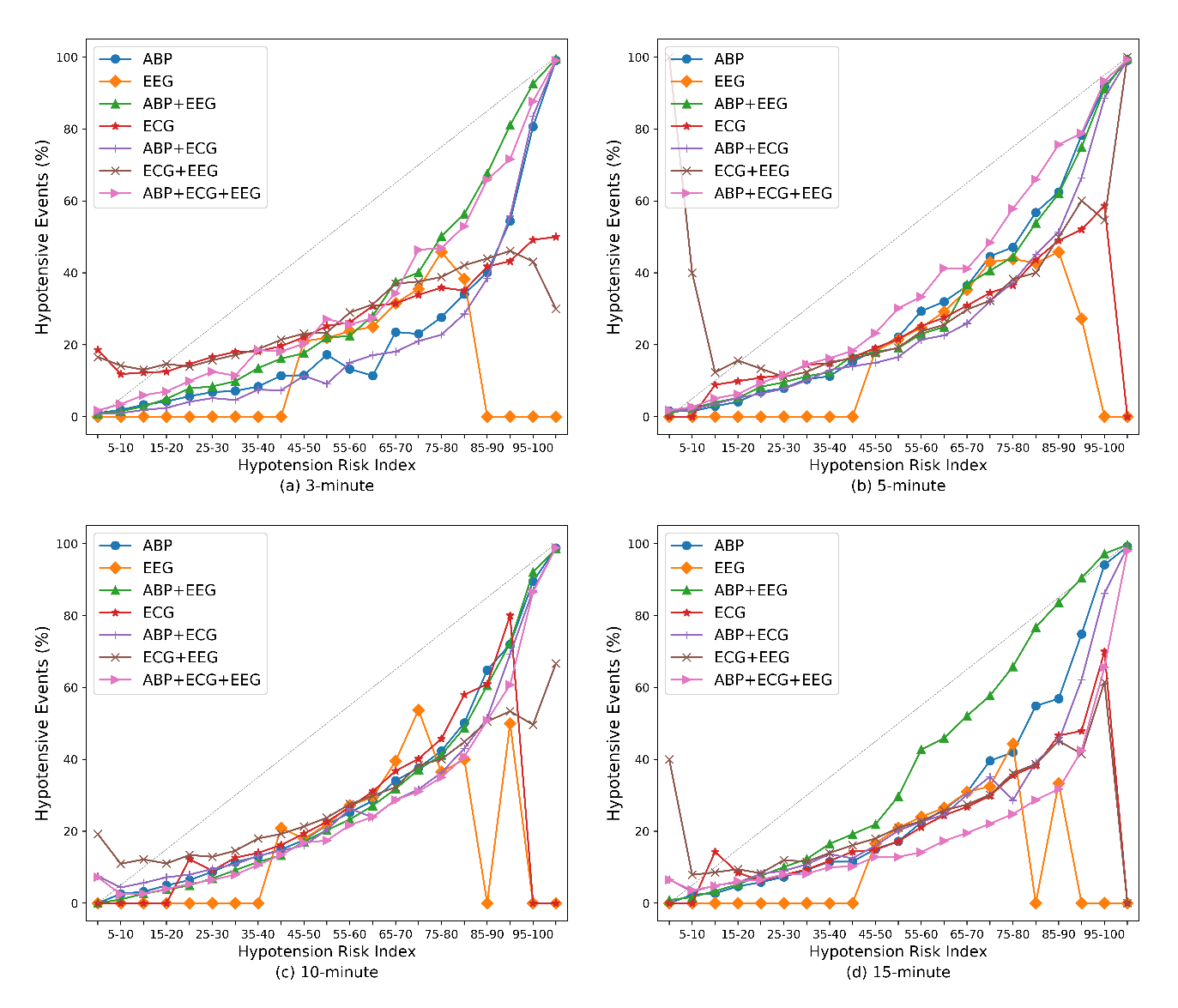


**Supplemental Figure 3.** Actual Occurrence of Hypotensive Events according to the Hypotension Risk Index for all combinations of waveforms. (a – d) Actual occurrence of hypotensive events according to the hypotension risk indices to predict the events 3, 5, 10, and 15 min before, respectively. ABP, arterial blood pressure; EEG, electroencephalogram; ECG, electrocardiogram.
