# Supplementary material for: Predicting intraoperative hypotension using deep learning with waveforms of arterial blood pressure, electroencephalogram, and electrocardiogram: Retrospective study
Source: PLoS One. 2022 Aug 9;17(8):e0272055. doi: 10.1371/journal.pone.0272055 (PMC9362925; doi:10.1371/journal.pone.0272055)
Supplement: S4 Fig — PPV, positive predictive value; NPV, negative predictive value; MAP, mean arterial pressure; ABP, arterial blood pressure. (DOCX) [file pone.0272055.s004.docx]

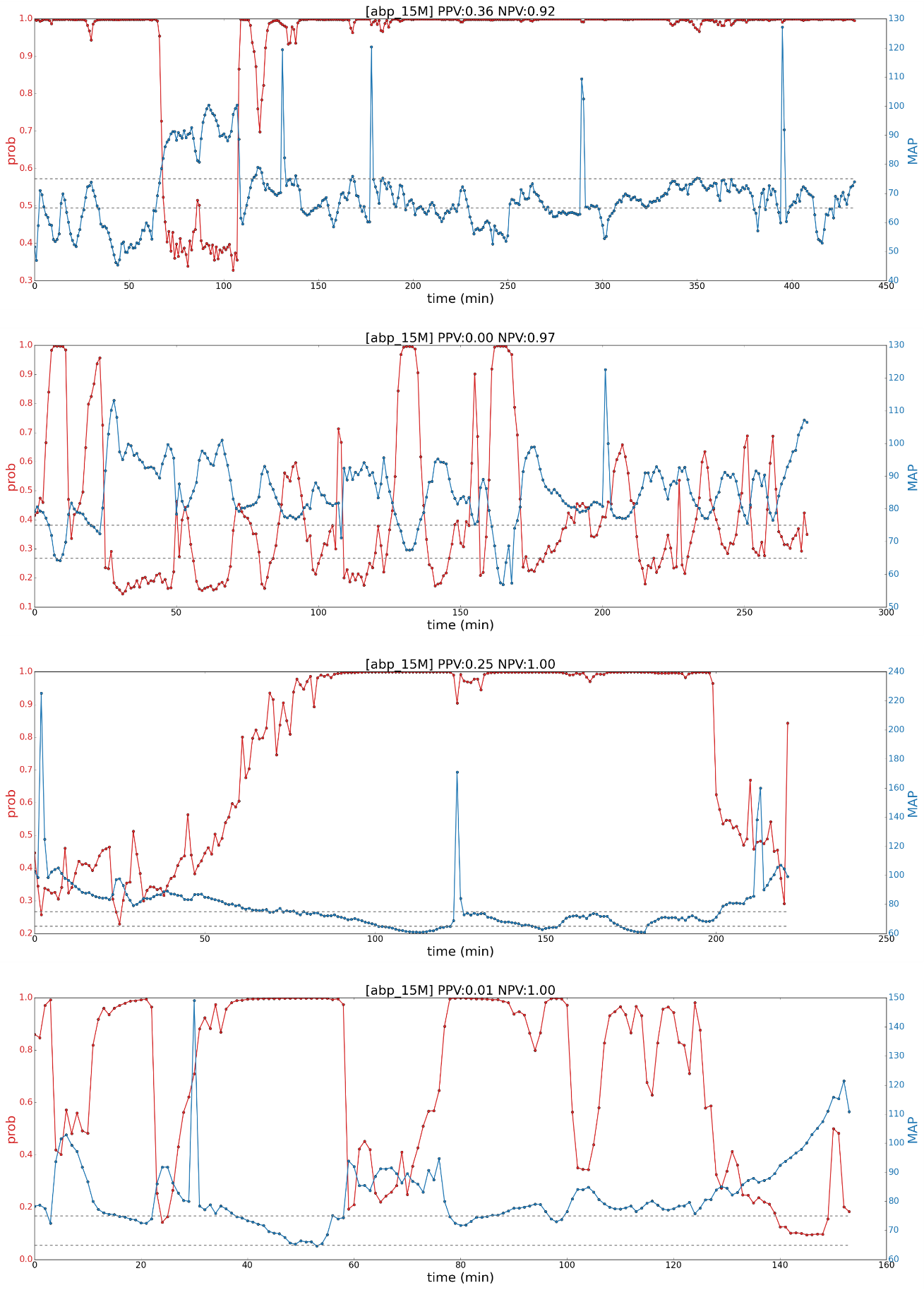


**Supplemental Figure 4.** The representative cases of prediction over the actual duration of a surgical procedure. PPV, positive predictive value; NPV, negative predictive value; MAP, mean arterial pressure; ABP, arterial blood pressure.
