# Supplementary material for: Predicting intraoperative hypotension using deep learning with waveforms of arterial blood pressure, electroencephalogram, and electrocardiogram: Retrospective study
Source: PLoS One. 2022 Aug 9;17(8):e0272055. doi: 10.1371/journal.pone.0272055 (PMC9362925; doi:10.1371/journal.pone.0272055)
Supplement: S1 Table — The models have 12 residual blocks. The model using ECG and model using blood pressure was set as same hyperparameter. The model using EEG has a different kernel size. (DOCX) [file pone.0272055.s005.docx]

**Supplemental Table1.** Hyperparameter setting for intraoperative hypotension prediction model. The models have 12 residual blocks. The model using ECG and model using blood pressure was set as same hyperparameter. The model using EEG has a different kernel size.

| **Residual block #** | **Kernel size (ECG, ABP / EEG)** | **Channel size** | **^a^Size down** | **Output size (ECG, ABP / EEG)** |
| --- | --- | --- | --- | --- |
| **Input data** |  |  |  | **30000 * 1 7680 * 1** |
| **1** | **15 /7** | **2** | **1** | **15000 * 2 3840 * 2** |
| **2** | **15 / 7** | **2** | **0** | **15000 * 2 3840 * 2** |
| **3** | **15 / 7** | **2** | **1** | **7500 * 2 1920 * 2** |
| **4** | **15 / 7** | **2** | **0** | **7500 * 2 1920 * 2** |
| **5** | **15 / 7** | **2** | **1** | **3750 * 2 960 * 2** |
| **6** | **15 / 7** | **4** | **0** | **3750 * 4 960 * 4** |
| **7** | **7 / 3** | **4** | **1** | **1875 * 4 480 * 4** |
| **8** | **7 / 3** | **4** | **0** | **1875 * 4 480 * 4** |
| **9** | **7 / 3** | **4** | **1** | **938 * 4 240 * 4** |
| **10** | **7 / 3** | **6** | **0** | **938 * 4 240 * 6** |
| **11** | **7 / 3** | **6** | **1** | **496 * 6 120 * 6** |
| **12** | **7 / 3** | **6** | **0** | **496 * 6 120 * 6** |

**^a^Size down: set 1 when length of data was halved by pooling**
