# Supplementary material for: Predicting intraoperative hypotension using deep learning with waveforms of arterial blood pressure, electroencephalogram, and electrocardiogram: Retrospective study
Source: PLoS One. 2022 Aug 9;17(8):e0272055. doi: 10.1371/journal.pone.0272055 (PMC9362925; doi:10.1371/journal.pone.0272055)
Supplement: S3 Table — (DOCX) [file pone.0272055.s007.docx]

**Supplemental Table 3.** Difference in model performance between the first event and recurrent events.

|  | First event | | Recurrent event | |
| --- | --- | --- | --- | --- |
| **Waveforms** | AUROC | AUPRC | AUROC | AUPRC |
| **Time to event: 3 min** |  |  |  |  |
| ABP | 0.955 | 0.889 | 0.988 | 0.994 |
| ECG | 0.575 | 0.295 | 0.664 | 0.788 |
| EEG | 0.568 | 0.262 | 0.592 | 0.750 |
| ABP + ECG | 0.955 | 0.897 | 0.987 | 0.994 |
| ABP + EEG | 0.956 | 0.906 | 0.990 | 0.995 |
| ECG + EEG | 0.622 | 0.335 | 0.673 | 0.795 |
| ABP + ECG + EEG | 0.951 | 0.897 | 0.984 | 0.993 |
| **Time to event: 5 min** |  |  |  |  |
| ABP | 0.885 | 0.786 | 0.975 | 0.988 |
| ECG | 0.590 | 0.301 | 0.687 | 0.805 |
| EEG | 0.598 | 0.316 | 0.603 | 0.752 |
| ABP + ECG | 0.884 | 0.768 | 0.974 | 0.988 |
| ABP + EEG | 0.835 | 0.793 | 0.976 | 0.989 |
| ECG + EEG | 0.631 | 0.369 | 0.677 | 0.801 |
| ABP + ECG + EEG | 0.829 | 0.701 | 0.974 | 0.988 |
| **Time to event: 10 min** |  |  |  |  |
| ABP | 0.846 | 0.704 | 0.945 | 0.975 |
| ECG | 0.604 | 0.284 | 0.694 | 0.815 |
| EEG | 0.632 | 0.321 | 0.605 | 0.762 |
| ABP + ECG | 0.792 | 0.665 | 0.930 | 0.970 |
| ABP + EEG | 0.852 | 0.718 | 0.948 | 0.976 |
| ECG + EEG | 0.602 | 0.301 | 0.677 | 0.802 |
| ABP + ECG + EEG | 0.711 | 0.560 | 0.946 | 0.976 |
