# Supplementary material for: Predicting intraoperative hypotension using deep learning with waveforms of arterial blood pressure, electroencephalogram, and electrocardiogram: Retrospective study
Source: PLoS One. 2022 Aug 9;17(8):e0272055. doi: 10.1371/journal.pone.0272055 (PMC9362925; doi:10.1371/journal.pone.0272055)
Supplement: S4 Table — (DOCX) [file pone.0272055.s008.docx]

**Supplemental Table 4.** The model performance on consecutively sampled waveforms with 1-minute interval over the entire surgical procedures in 100 randomly selected cases

|  | ABP_3min | | ABP_15min | | ABP+EEG_3min | | ABP+EEG_15min | |
| --- | --- | --- | --- | --- | --- | --- | --- | --- |
| **Threshold** | **PPV** | **NPV** | **PPV** | **NPV** | **PPV** | **NPV** | **PPV** | **NPV** |
| 0.1 | 0.142 | 0.964 | 0.118 | 0.988 | 0.128 | 0.950 | 0.118 | 0.996 |
| 0.3 | 0.165 | 0.967 | 0.122 | 0.908 | 0.142 | 0.955 | 0.119 | 0.967 |
| 0.5 | 0.177 | 0.967 | 0.127 | 0.905 | 0.156 | 0.962 | 0.122 | 0.919 |
| 0.7 | 0.192 | 0.968 | 0.136 | 0.905 | 0.171 | 0.962 | 0.126 | 0.909 |
| 0.9 | 0.213 | 0.965 | 0.145 | 0.899 | 0.191 | 0.956 | 0.134 | 0.903 |

**PPV, positive predictive value; NPV, negative predictive value.**
