# Supplementary material for: Predicting intraoperative hypotension using deep learning with waveforms of arterial blood pressure, electroencephalogram, and electrocardiogram: Retrospective study
Source: PLoS One. 2022 Aug 9;17(8):e0272055. doi: 10.1371/journal.pone.0272055 (PMC9362925; doi:10.1371/journal.pone.0272055)
Supplement: S5 Table — (DOCX) [file pone.0272055.s009.docx]

**Supplemental Table 5.** Comparison of test dataset between the main and post hoc analysis

| Test (main result) | | Test (post hoc) | |
| --- | --- | --- | --- |
| Event samples (cases) | Non-event samples (cases) | Event samples (cases) | Non-event samples (cases) |
| 8,403  (2,925) | 27,204  (3,103) | 56,461  (2,925) | 27,204  (3,103) |
| 8,346  (2,953) | 27,358  (3,105) | 55,647  (2,953) | 27,358  (3,105) |
| 8,026  (2,877) | 27,293  (3,084) | 56,213  (2,877) | 27,293  (3,084) |
| 7,153  (2,703) | 28,099  (3,137) | 50,358  (2,703) | 28,099  (3,137) |
