# Supplementary material for: Predicting intraoperative hypotension using deep learning with waveforms of arterial blood pressure, electroencephalogram, and electrocardiogram: Retrospective study
Source: PLoS One. 2022 Aug 9;17(8):e0272055. doi: 10.1371/journal.pone.0272055 (PMC9362925; doi:10.1371/journal.pone.0272055)
Supplement: S6 Table — P-values were calculated with DeLong’s method and corrected using Bonferroni’s method. (DOCX) [file pone.0272055.s010.docx]

**Supplemental Table 6.** Post hoc analysis: statistical significance of comparison of models using different combinations of waveforms. P-values were calculated with DeLong’s method and corrected using Bonferroni’s method.

| Time to event: 3 min | | | | | | |
| --- | --- | --- | --- | --- | --- | --- |
| Waveforms | ABP | ECG | EEG | ABP+ECG | ABP+EEG | ECG+EEG |
| ECG | <0.001 |  |  |  |  |  |
| EEG | <0.001 | <0.001 |  |  |  |  |
| ABP+ECG | >0.999 | <0.001 | <0.001 |  |  |  |
| ABP+EEG | <0.001 | <0.001 | <0.001 | <0.001 |  |  |
| ECG+EEG | <0.001 | <0.001 | <0.001 | <0.001 | <0.001 |  |
| ABP+ECG+EEG | <0.001 | <0.001 | <0.001 | <0.001 | <0.001 | <0.001 |
| Time to event: 5 min | | | | | | |
| Waveforms | ABP | ECG | EEG | ABP+ECG | ABP+EEG | ECG+EEG |
| ECG | <0.001 |  |  |  |  |  |
| EEG | <0.001 | <0.001 |  |  |  |  |
| ABP+ECG | 0.067 | <0.001 | <0.001 |  |  |  |
| ABP+EEG | 0.015 | <0.001 | <0.001 | <0.001 |  |  |
| ECG+EEG | <0.001 | <0.001 | <0.001 | <0.001 | <0.001 |  |
| ABP+ECG+EEG | 0.047 | <0.001 | <0.001 | >0.999 | <0.001 | <0.001 |
| Time to event: 10 min | | | | | | |
| Waveforms | ABP | ECG | EEG | ABP+ECG | ABP+EEG | ECG+EEG |
| ECG | <0.001 |  |  |  |  |  |
| EEG | <0.001 | <0.001 |  |  |  |  |
| ABP+ECG | <0.001 | <0.001 | <0.001 |  |  |  |
| ABP+EEG | <0.001 | <0.001 | <0.001 | <0.001 |  |  |
| ECG+EEG | <0.001 | <0.001 | <0.001 | <0.001 | <0.001 |  |
| ABP+ECG+EEG | 0.004 | <0.001 | <0.001 | <0.001 | <0.001 | <0.001 |
| Time to event: 15 min | | | | | | |
| Waveforms | ABP | ECG | EEG | ABP+ECG | ABP+EEG | ECG+EEG |
| ECG | <0.001 |  |  |  |  |  |
| EEG | <0.001 | <0.001 |  |  |  |  |
| ABP+ECG | <0.001 | <0.001 | <0.001 |  |  |  |
| ABP+EEG | <0.001 | <0.001 | <0.001 | <0.001 |  |  |
| ECG+EEG | <0.001 | <0.001 | <0.001 | <0.001 | <0.001 |  |
| ABP+ECG+EEG | <0.001 | <0.001 | <0.001 | <0.001 | <0.001 | <0.001 |
